# Supplementary material for: The Effect of Precipitation pH on Protein Recovery Yield and Emulsifying Properties in the Extraction of Protein from Cold-Pressed Rapeseed Press Cake
Source: Molecules. 2022 May 5;27(9):2957. doi: 10.3390/molecules27092957 (PMC9104397; doi:10.3390/molecules27092957)
Supplement: Supplementary file 1 [file molecules-27-02957-s001.zip › molecules-1696251-supplementary.pdf]

Supplementary Materials

The Effect of Precipitation pH on Protein Recovery Yield and Emulsifying Properties in the Extraction of Protein from Cold-pressed Rapeseed Press Cake

Cecilia Ahlström [1,*], Johan Thuvander [1], Marilyn Rayner [1], María Matos [2], Gemma Gutiérrez [2] and Karolina Östbring [1]

[1] Department of Food Technology Engineering and Nutrition, Lund University, Naturvetarvägen 12, 223 62 Lund, Sweden; johan.thuvander@food.lth.se (J.T.); marilyn.rayner@food.lth.se (M.R.); karolina.ostbring@food.lth.se (K.Ö.)
[2] Department of Chemical and Environmental Engineering, University of Oviedo, Julián Clavería 8, 33006 Oviedo, Spain; matosmaria@uniovi.es (M.M); gutierrezgemma@uniovi.es (G.G)
* Correspondence: cecilia.ahlstrom@food.lth.se (C.A.)

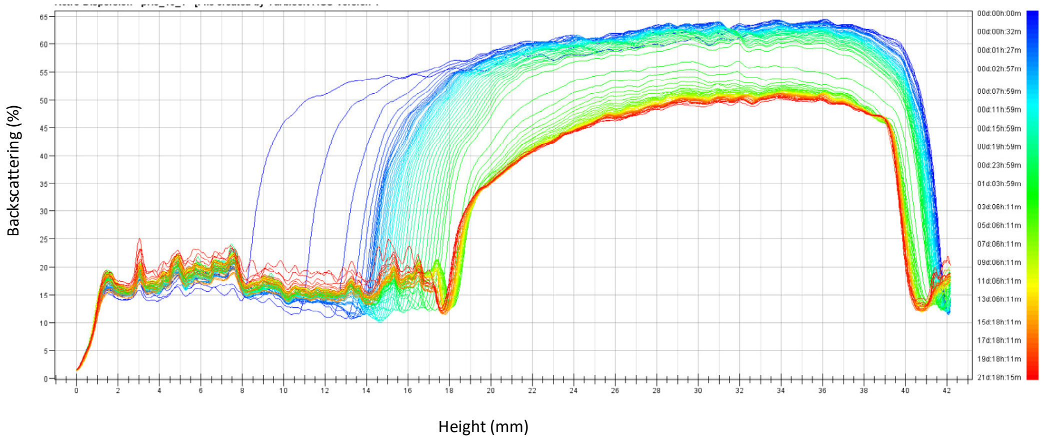

(a) pH 3.0

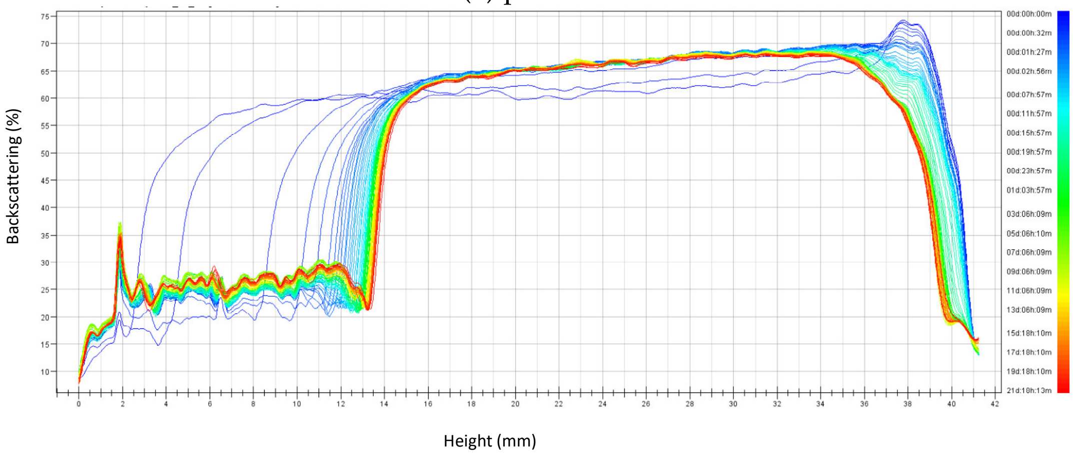

(b) pH 3.5

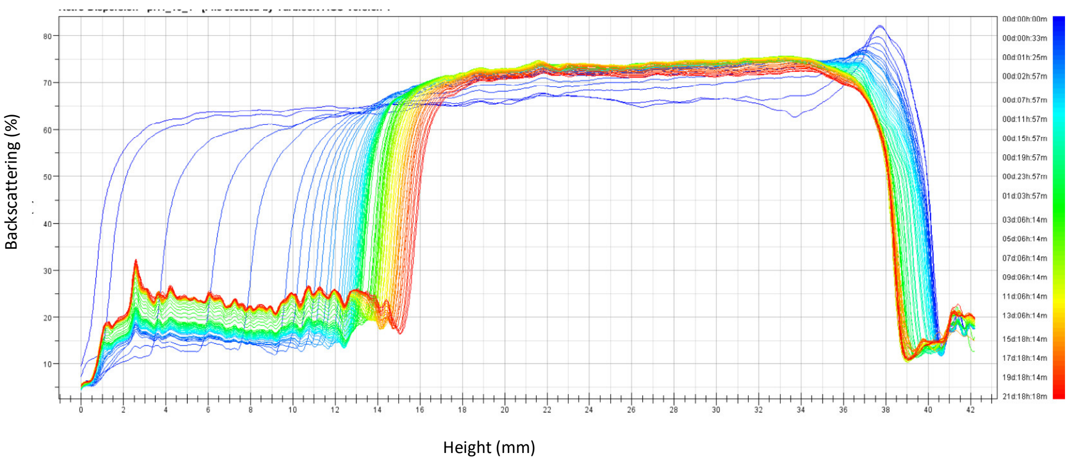

(c) pH 4.0

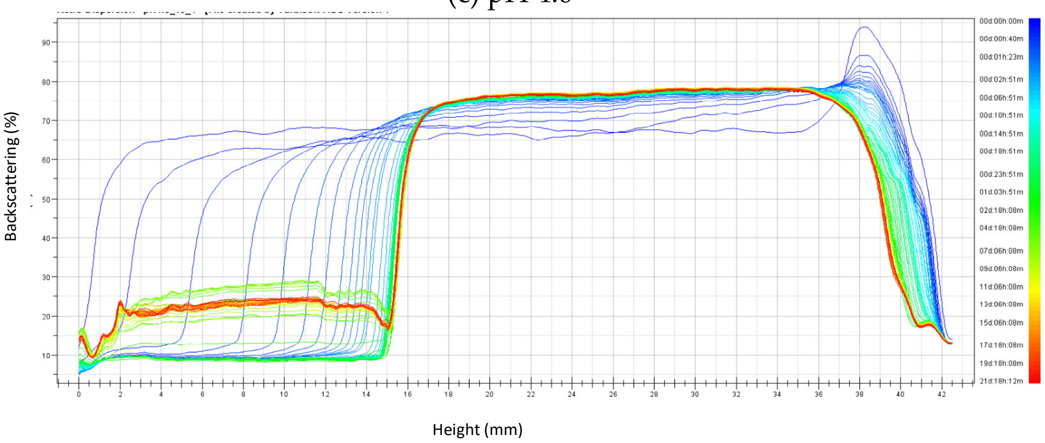

(d) pH 4.5

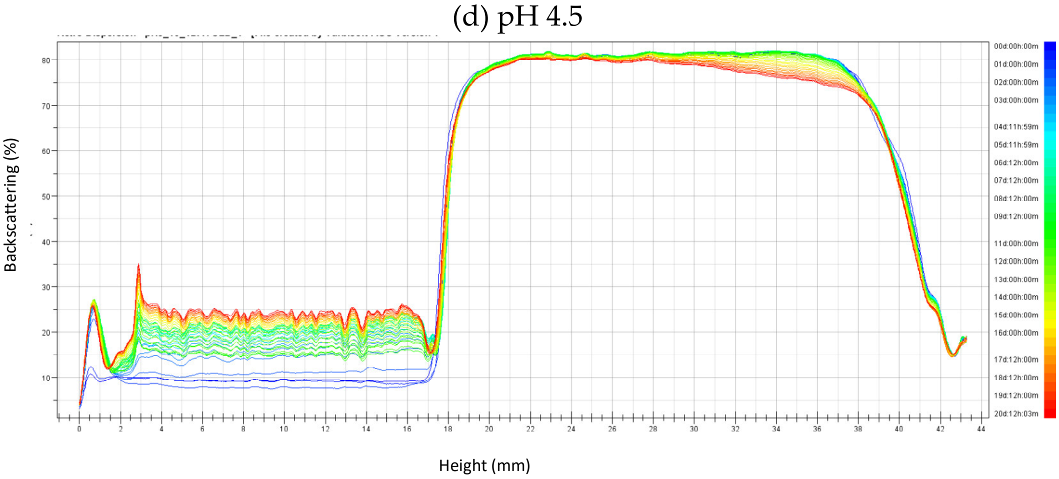

(e) pH 5.0

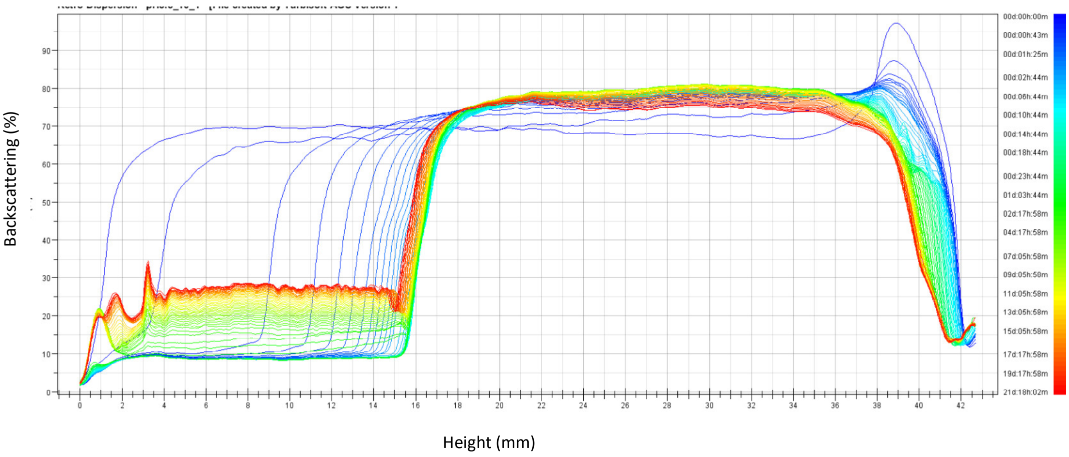

(f) pH 5.5

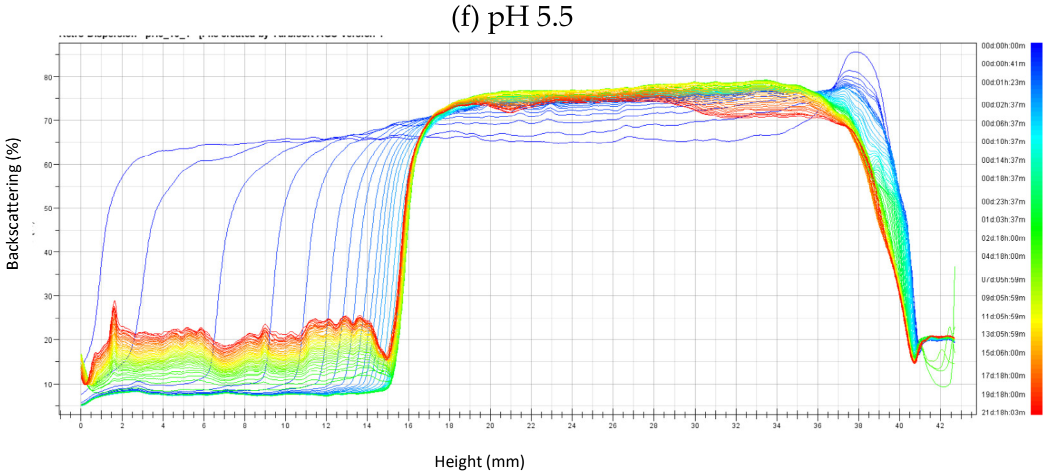

(g) pH 6.0

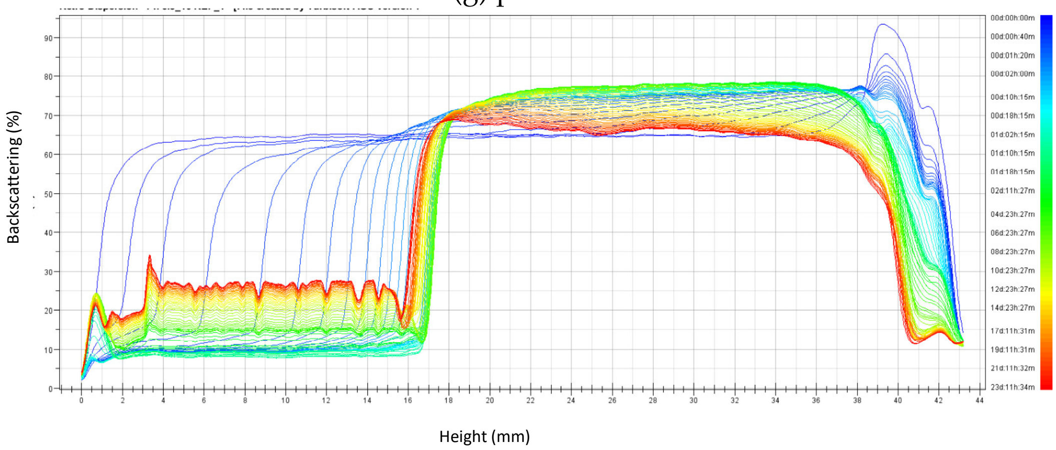

(h) pH 6.5

Figure S1. Backscattering profiles of the emulsions prepared with proteins stabilized at several pHs.

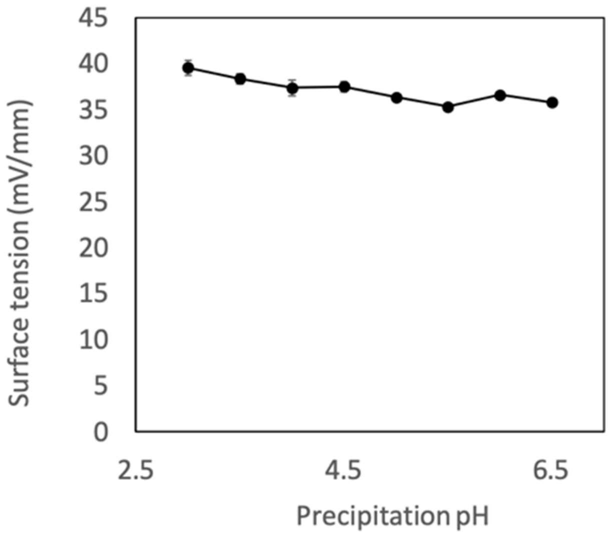

Figure S2. Surface tension measurements of protein solutions with proteins precipitated at several pHs.
